# Comparison of the MicroRNA Expression Profiles of Male and Female Avian Primordial Germ Cell Lines

**DOI:** 10.1155/2018/1780679

**Published:** 2018-07-10

**Authors:** Bence Lázár, Mahek Anand, Roland Tóth, Eszter Patakiné Várkonyi, Krisztina Liptói, Elen Gócza

**Affiliations:** ^1^Animal Biotechnology Department, NARIC, ABC, Gödöllő, Hungary; ^2^Research Centre for Farm Animal Gene Conservation, Gödöllő, Hungary; ^3^SZIU, Doctoral School of Animal Husbandry Science, Gödöllő, Hungary

## Abstract

Primordial germ cells (PGCs) are the precursors of adult germ cells, and among the embryonic stem-like cells in the bird embryo, only they can transmit the genetic information to the next generation. Despite the wide range of applications, very little is known about the mechanism that governs primordial germ cell self-renewal and differentiation. As a first step, we compared 12 newly established chicken PGC lines derived from two different chicken breeds, performing CCK-8 proliferation assay. All of the lines were derived from individual embryos. A significant difference was found among the lines. As microRNAs have been proved to play a key role in the maintenance of pluripotency and the cell cycle regulation of stem cells, we continued with a complex miRNA analysis. We could discover miRNAs expressing differently in PGC lines with high proliferation rate, compared to PGC lines with low proliferation rate. We found that gga-miR-2127 expresses differently in female and male cell lines. The microarray analysis also revealed high expression level of the gga-miR-302b-3p strand (member of the miR-302/367 cluster) in slowly proliferating PGC lines compared to the gga-miR-302b-5p strand. We confirmed that the inhibition of miR-302b-5p significantly increases the doubling time of the examined PGC lines. In conclusion, we found that gga-miR-181-5p, gga-miR-2127, and members of the gga-miR-302/367 cluster have a dominant role in the regulation of avian primordial germ cell proliferation.

## 1. Introduction

The avian embryo provides an excellent model system for developmental and cell biology. It has been widely used in early embryogenesis, toxicology, and stem cell studies [1–3].

Primordial germ cells (PGCs) are the precursors of adult germ cells and have remarkable potential in developmental biology-, disease modelling-, and drug development-related research applications [4]. They are easily accessible from the embryonic blood and are suitable for genome preservation (gene banks) [5].

Furthermore, the genetic modification of PGCs (via TALEN or CRISPR/CAS9) is the most efficient way to create genetically engineered bird models for basic and applied purposes [6, 7].

Despite the wide range of applications, very little is known about the mechanisms and miRNAs that govern primordial germ cell self-renewal and differentiation. MicroRNAs are endogenously expressed small noncoding RNAs. They tend to regulate and express in multiple biological processes such as cell growth, differentiation, apoptosis, and development [8].

The main function of the mature miRNAs is posttranscriptional regulation of mRNAs. The miRNAs have a specific sequence known as the seed sequence that binds to the 3′UTR of the mRNA, inhibiting the expression of the mRNA and preventing protein translation [9].

An emerging field in molecular biology is to investigate the potential role of miRNAs in regulating the pluripotency of stem and germ cells such as embryonic stem cells (ESCs), induced pluripotent stem cells (iPSCs), and primordial germ cells (PGCs). The role of stem cell-specific miRNAs was initially studied in mouse [10]. In mouse ESCs, knocking out the enzymes Drosha and Dicer resulted in lower proliferation and slower cell cycle rate [11].

Since then, numerous studies were carried out to characterize the most important miRNAs taking part in controlling the pluripotency and self-renewal of ESCs, iPSCs, and PGCs [12–14]. The gga-miR-302 cluster is evolutionary conserved and vertebrate-specific [15]. In chicken, the members of this cluster are miR-302a, miR-302b-5p, miR-302b-3p, miR-302c-3p, miR-302c-5p, miR-302d, and miR-367. This cluster is cited to be stem cell-specific and to play an important role in cell cycle regulation, especially in promoting the transition from the G1 to S phase. gga-miR-302a has been found to be a regulator of the expression of pluripotency factor OCT4. However, the two strands of miR-302b, miR-302b-5p and miR-302b-3p, tend to show concordant dysregulation [16]. In gastric cancer stem cell lines, miR-302b-3p attenuates proliferation and acts as a tumour suppressor via the AKT signalling pathway [17]. miR-302b-5p acts as a promoter of proliferation via MAPK signalling [18]. Similarly, miR-181a-5p and miR-2127 are potential tumour suppressor miRNAs as miR-181a-5p inhibits cell proliferation via MMP14, which is a matrix metalloproteinase enzyme. In chickens, the *MMP14* gene is a potential target of gga-miR-2127. It is speculated that gga-miR-2127 regulates proliferation via the proteolytic cleavage pathway of these matrix proteins [19].

In this article, we will present a detailed study on the proliferation and developmental properties of male and female chicken PGC lines and furthermore analyse the miRNA expression in four cell lines with a microarray assay. The main aim of this complex microarray study was to get an overview about the miRNAs expressed in the male and female chicken PGCs and based on the obtained data to select specific miRNAs for further analysis.

## 2. Materials and Methods

### 2.1. Experimental Animals and Animal Care

Animals were kept according to the standard rules of Hungarian Animal Protection Law (1998. XXVIII). Permission for experimental animal research at the Research Centre for Farm Animal Gene Conservation (Gödöllő, Hungary) was provided by the National Food Chain Safety Office, Animal Health and Animal Welfare Directorate, Budapest. The procedures for animal management and embryo manipulation followed the standard operating protocols of our laboratory and were approved by the NARIC Agricultural Biotechnology Institute. The Partridge colour chicken breed was kept in the Research Centre for Farm Animal Gene Conservation (Gödöllő, Hungary). The GFP-expressing White Leghorn breed was identical with the one that McGrew and colleagues described earlier [20].

### 2.2. Establishment of PGC Lines

Eggs were collected and incubated before the experiment. PG cell lines were derived by adding 1 *μ*l blood isolated from HH stage 14–17 embryos to a selective PGC media—developed by McGrew and colleagues [21]—to eliminate the cellular elements of the blood and support the division of the PG cells. One-third of the medium was changed every 2 days. A line was considered successfully established, if the cell number reached 5.0 × 10^4^ until the end of the 3rd week. All the examined PGC lines were derived from individual embryos.

### 2.3. DNA Isolation and Sex Determination

For isolating the DNA, High Pure PCR Template Preparation Kit (Roche Diagnostics, US) was used according to the manufacturer's instruction. Samples were stored at −20°C until the next step. The sex of the donor embryos and the established PGC lines were determined with the P2–P8 primer set as described before by Griffiths and colleagues [22] (Supplementary Table 1). The isolated DNA was diluted to 25 ng/*μ*l concentration for PCR reaction and gel electrophoresis. MyTaq Red Mix was used for the reaction (Bioline Reagents Ltd., UK). The PCR products were then separated by electrophoresis, using 3% agarose gel stained with ethidium bromide at 100 V for 1.5–2.0 hours. The DNA bands were then visualized under UV illumination and photographed.

### 2.4. RNA Isolation, Synthesis of cDNA, and Quantitative Real-Time PCR

Total RNA from the established PGC lines was isolated using TRI Reagent® (MRC, UK) [23] following the instructions of the manufacturer. The concentration of RNA was determined by NanoDrop Spectrophotometer, and the samples were stored at −70°C until later use. The extracted RNA samples were reverse transcribed into cDNA with High Capacity cDNA Reverse Transcription Kit following the instructions of the manufacturer (Applied Biosystems, Life Technologies, Carlsbad, US). RT Master Mix was used for cDNA writing. The cDNA was stored at −20°C. The synthesized cDNA was then used for quantitative real-time PCR. SYBR Green PCR Master Mix was applied for the qPCR as a double-stranded fluorescent DNA-specific dye according to the manufacturer's instructions (Applied Biosystems, Life Technologies, Carlsbad, US) (Supplementary Table 1). For each gene examined, three parallels were analysed, fluorescence emission was detected, and relative quantification was calculated with the GenEx program (MultiD, SE).

### 2.5. Immunostaining of PGCs

Isolated PGCs were fixed with 4% PFA for 10 minutes. After washing with PBS (three times, five minutes each), cells were permeabilised with 0.5% Triton X-100 (Merck Millipore, US) for 5 minutes. After washing with PBS, to minimize nonspecific binding of antibodies, the fixed cells were blocked for 45 minutes with a blocking buffer containing PBS with 1% (*v*/*v*) BSA. Then, cells were washed three times with PBS and were incubated with each of the primary antibodies including mouse anti-SSEA-1 (1 : 10, Developmental Studies Hybridoma Bank, US) and rabbit anti-VASA (1 : 1000; kindly provided by Bertrand Pain, Lyon, France). After incubation overnight in the primary antibody solution in a humid chamber at 4°C, the cells were washed three times with PBS. Then, cells were incubated with the secondary antibodies, donkey anti-mouse IgM FITC Cy3 (1 : 400, Jackson ImmunoResearch, USA), donkey anti-rabbit IgG FITC (1 : 400, Jackson ImmunoResearch, USA), and donkey anti-rabbit IgG conjugated to Alexa 555 (1 : 400, Molecular Probes Inc., USA), in a dark humid chamber for 1 hour at room temperature. After washing with PBS, the nucleus was stained with TO-PRO®-3 stain (1 : 500, Molecular Probes Inc., US), which is a far-red fluorescent (642/661) nuclear and chromosome counterstain. Coverslips were mounted on the slide with the application of 20 *μ*l VECTASHIELD® Mounting Media (Vector Laboratories Inc., US) and analysed by confocal microscopy (TCS SP8, Leica). Negative controls were stained only with the secondary antibody.

### 2.6. Cell Proliferation Assay

After 1 day of culture, half of the medium was carefully replaced with fresh medium containing diluted CCK-8 reagent (1 : 10 final concentration, Dojindo Laboratories, Japan) and incubated for 3 hours at 37°C. The product of the CCK-8 reaction was quantified by measuring absorbance (OD) at 450 nm using a CLARIOstar® Microplate Reader (BMG Labtech, US). Three 96-well plates (as biological replicates), with 6-6 parallel wells, were prepared for each condition.

### 2.7. Doubling Time Calculation

The doubling time is the time required for a culture to double in number. We calculated the doubling time using the following formula, Gr (growth rate) = ln(*N*(*t*)/*N*(0))/*t*, where *N*(*t*) is the number of cells at time *t* and *N*(0) is the number of cells at time *t* = 0 (*t* expressed in days). Therefore, doubling time = ln(2)/growth rate (Gr). The doubling rate is inversely proportional to the proliferation rate.

### 2.8. MicroRNA Microarray Assay

For analysing the miRNA expression patterns, the samples were sent to LC Sciences, Houston, TX, USA. Microarray assay kit was performed by the LC Sciences Company. This assay is based on *μ*Paraflo® microchip technology. The process started with the 3′-extension with a poly A-tail of 4 to 8 *μ*g of total RNA using poly(A) polymerase. An oligonucleotide tag was later ligated to the poly(A) tail for fluorescent staining. Following this, hybridization was performed overnight on the *μ*Paraflo microfluidic chip using a microcirculation pump (Atactic Technologies, Houston, TX, USA). The probes were designed based upon the miRBase 21 database (Supplementary Table 6). The probes consisted of a coding sequence and a long spacer. The coding sequence is complementary to the mature miRNA sequence and contains chemical modification for enhancing the specificity and sensitivity of detection, as well as for balancing the melting temperature of probes for hybridization. The spacer sequence is nonnucleotide-specific and is to prevent noncomplementarity binding. The probe synthesis is in situ and based on the principle of light PGA lithography. Following hybridization, the tag-conjugating dye Cy3 was circulated throughout the microfluidic chip. The fluorescent images were obtained using the Axon GenePix 4000B Microarray Scanner (Molecular Devices, Sunnyvale, CA) and digitized by the Array-Pro image analysis software (Media Cybernetics, Rockville, MD). The data were analysed by first subtracting the background and then normalizing the signals using a LOWESS filter (locally weighted regression) (Supplementary Tables 2–5).

### 2.9. Transfection of Chicken PGC Lines with Anti-miR-302b-3p and Anti-miR-302b-5p Inhibitors

Two weeks before the transfection, chicken FS101 and FS111 PS PGC lines were thawed. After two weeks in the culture, PGCs were collected by centrifugation and plated to 96-well plates (1000 cells/well). Next day, the cells were transfected with anti-miR-302b-5P and anti-miR-302b-3P vectors (at 100 nM final concentration) (Supplementary Table 1) (Applied Biosystems, Life Technologies, Carlsbad, US) using a siPORT™ (Applied Biosystems, Life Technologies, Carlsbad, US) transfection agent according to the manufacturer's instructions. Three 96-well plates (as biological replicates), with 6-6 parallel wells, were prepared for each condition. The proliferation rate of treated and control cells was measured using CCK-8 reagent (1 : 10, Dojindo Laboratories, Japan) every day, for 3 days. For detailed RNA expression analysis, cells were harvested in lysis buffer of RNAqueous®-Micro Kit (Applied Biosystems, Life Technologies, Carlsbad, US) 48 hours following the transfection.

### 2.10. Statistical Analysis

All data were analysed by R Studio (version 1.0.136), R (version R-3.2.2.) and GenEx (version 6.0) (*p* < 0.05 was considered significant (^∗^ *p* < 0.05, ^∗∗^ *p* < 0.01, and ^∗∗∗^ *p* < 0.001)).

Individual PGC samples served as the experimental unit for all statistical analyses.

Agglomerative hierarchical clustering was used to examine the similarities/dissimilarities between the PGC lines. The box plots represent descriptive statistical parameters which were calculated from the expression datasets for each marker. Welch's *t*-tests were performed between the groups in case of each variable.

Correlation coefficient (1 to −1) and the significance of the relationship (^∗∗∗^ *p* < 0.001, ^∗∗^ *p* < 0.01, ^∗^ *p* < 0.05, and .*p* < 0.1) were calculated for each pair of the markers. Bivariate scatter plots are also shown with a fitted line on every marker combination.

The expression or repression of the target gene relative to the internal control gene in each sample was calculated with GenEx 6.0 program (MultiD, SE) using 2^−ΔΔCt^ where ΔCt = Ct target gene − Ct internal control and ΔΔCt = ΔCt test sample − ΔCt control sample.

Statistical differences between the examined groups were assessed by *t*-test using the GenEx 6.0 software.

## 3. Results

### 3.1. Establishment of the Chicken PGC Lines

In our manuscript, we are presenting a detailed study on the proliferation and developmental properties of 12 chicken PGC lines. All the lines were derived from individual embryos.

**Figure 1:**
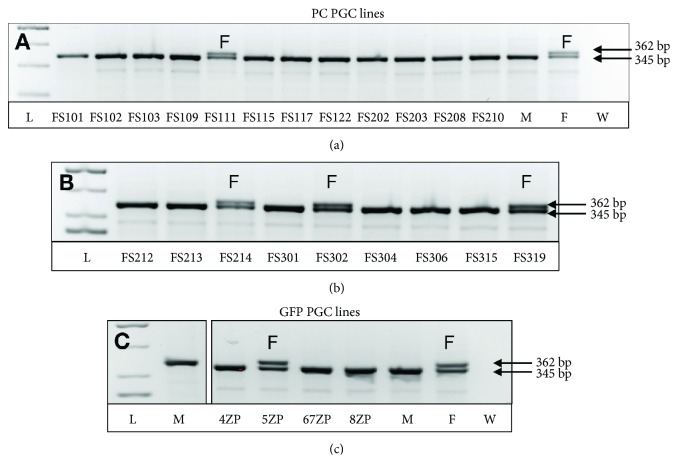
Sex determination in established PGC lines. The sex determination was performed by using the P2–P8 primer set (Supplementary Table 1). The size of the PCR products was 362 bp and 345 bp. In the case of the female cell lines, two bands were detected. (a) Analysis of PC PGC lines: only the FS111 PGC lines is female. (b) Analysis of PC PGC lines: FS214, FS302, and FS319 lines are females. (c) Analysis of GFP PGC lines: only the 5ZP line is female. L: ladder; M: male control sample; F: female control sample; W: nuclease-free water. Arrows indicated the size of the fragments. PC PGC lines: Partridge colour Hungarian chicken embryo-derived primordial germ cell lines; GFP PGC lines: green fluorescent protein- (GFP-) expressing transgenic chicken embryo-derived primordial germ cell lines.

Recently, we established 21 PGC lines from Partridge colour Hungarian chicken breed (PC lines) (Table 1(a)) and 10 from GFP-expressing White Leghorn (GFP lines) (Table 1(b)) chicken breed. Significantly less female lines were derived than males. Among the 21 PC lines, only 4 (19%), in case of the 10 GFP lines, only 2 (20%) female PGC lines were detected by using sex PCR (Figure 1).

As a next step, we characterized the *in vitro* and *in vivo* developmental potential of the newly established cell lines.

### 3.2. Characterisation of the Chicken PGC Lines

**Figure 2:**
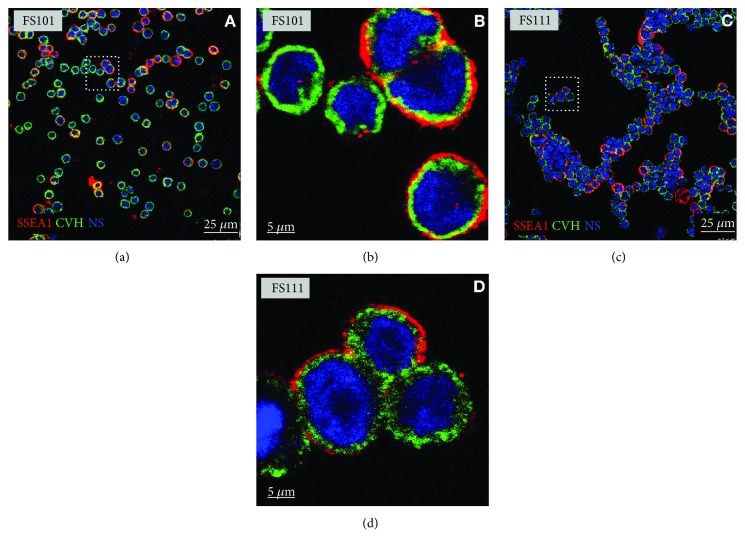
Immunofluorescence staining of FS101 and FS111 PC PGCs. (a) Confocal images of SSEA-1- (red) and CVH- (green) stained FS101 PGCs. (b) Higher magnification of FS101 cells (white square indicates the cells on (a)). (c) Confocal images of SSEA-1- (red) and CVH- (green) stained FS111 PGCs. (d) Higher magnification of the FS111 cells (white square indicates the cells on (c)). For nuclear staining (NS), we used TO-PRO-3 (blue). Scale bars: 25 *μ*m (a, c) and 5 *μ*m (b, d). PC PG cells: Partridge colour Hungarian chicken embryo-derived primordial germ cells.

**Figure 3:**
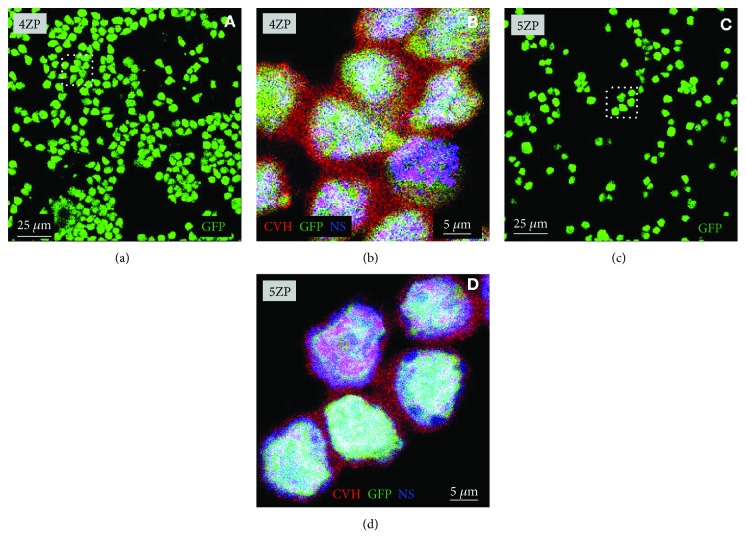
Immunofluorescence staining of 4ZP and 5ZP GFP PGCs. (a) Confocal images of GFP-expressing (green) 4ZP PGCs. (b) Higher magnification of 4ZP PGCs showing CVH expression (CVH (red), GFP (green), and NS (blue)) (white square indicates the cells on (a)). (c) Confocal images of GFP-expressing (green) 5ZP PGCs. (d) Higher magnification of 5ZP PGCs showing CVH expression (CVH (red), GFP (green), and NS (blue)) (white square indicates the cells on (c)). For nuclear staining (NS), we used TO-PRO-3 (blue). Scale bars: 25 *μ*m (a, c) and 5 *μ*m (b, d). GFP PG cells: green fluorescent protein- (GFP-) expressing transgenic chicken embryo-derived primordial germ cells.

First, we examined the CVH and SSEA-1 expression in PGC lines by immunofluorescent staining (Figures 2 and 3). All the established PGC lines highly expressed CVH and SSEA-1.  Figure 2 demonstrates the immunostaining of two PC lines (FS101 and FS111), while Figure 3 displays GFP and CVH expression in two GFP expressing PGC lines (4ZP and 5ZP). All cells in GFP PGC lines expressed CVH (Figure 3). To check the in vivo developmental properties of PGCs, we injected them back into 3-day-old recipient embryos. We investigated the integration ratio of injected PGCs in 7-day-old embryo's gonads (Table 2). Using PC embryo-derived PG cells, 50 percent of the injected embryos (Table 2(a)) contained donor-derived germ cells, while in the case of GFP PGC lines, we got 20% chimeric gonads (Table 2(b)).

These preliminary experiments revealed that our PGC lines are able to form viable germ cells, but the fact that we got less chimeric gonads in the case of examined GFP cell lines revealed that there is a difference in the developmental properties among them.

### 3.3. Comparison of the Stem Cell- and Germ Cell-Specific Properties of Chicken PGC Lines

**Figure 4:**
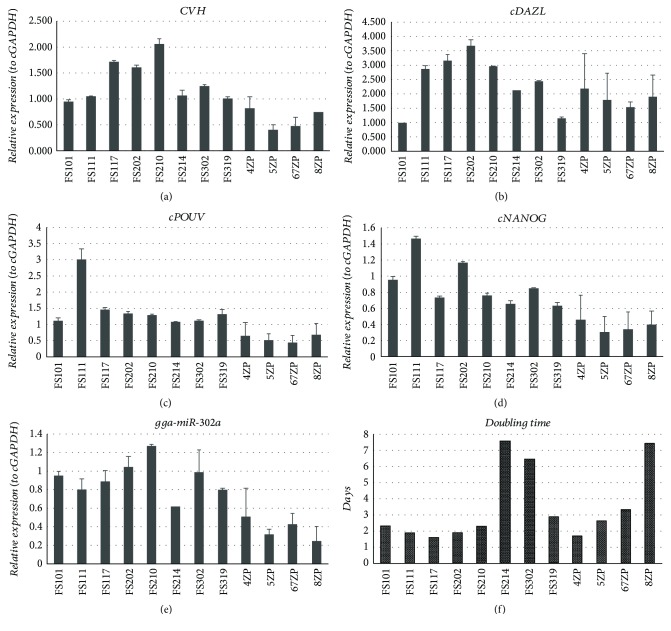
Stem cell- and germ cell-specific marker expression and the doubling time analysis in PC and GFP PGC lines. 4 female PC PGC lines (FS111, FS214, FS302, and FS319), 4 male PC PGC lines (FS101, FS117, FS202, and FS210), 1 female GFP PGC line (5ZP), and 3 male GFP PGC lines (4ZP, 67ZP, and 8ZP) were investigated in detail. We examined two biological samples as repeats. 3 parallel samples were used at qPCR runs. We analysed the CVH (a), cDAZL (b), cPOUV (c), and cNANOG (d) (using cGAPDH as reference gene) and the expression of a stem cell-specific miR-302a (relative to miR-92 as reference gene). Gene expression values were calculated using GenEx software relative to the FS101 sample in each case. Supplementary Table 1 contains detailed information of the analysed genes and the sequences of the primers used at qPCR runs. (f) Furthermore, the proliferation rate of the cell lines was measured on 3 different days using CCK-8 proliferation assay. We used three 96-well plates with 6-6 parallel wells. The doubling time was calculated according to the measured optical densities (OD). PC PGC lines: Partridge colour Hungarian chicken embryo-derived primordial germ cell lines. GFP PGC lines: green fluorescent protein- (GFP-) expressing transgenic chicken embryo-derived primordial germ cell lines.

We compared 4 female PC PGC cell lines (FS111, FS214, FS302, and FS319), 4 male PC PGC lines (FS101, FS117, FS202, and FS210), 1 female GFP PGC line (5ZP), and 3 male GFP PGC lines (4ZP, 67ZP, and 8ZP). We analysed two biological repeats at each cell line. At qPCR runs, we used 3 parallel samples. The *CVH*, *cDAZL*, *cPOUV*, and *cNANOG* expression and the miR-302a stem cell-specific miRNA expression were examined by qPCR (Figure 4, Supplementary Table 1). Furthermore, the proliferation rate of the cell lines was measured on 3 different days using CCK-8 proliferation assay. We used three 96-well plates (as biological parallels) with 6-6 parallel wells. The doubling time was calculated from the measured optical densities (OD) (Figure 4).

Results were analysed using R Studio program. We found differences in the expression level of stem cell- and germ cell-specific markers, but there was a significant difference only in the proliferation rate among the examined eight cell lines (Figure 4(f)).

**Figure 5:**
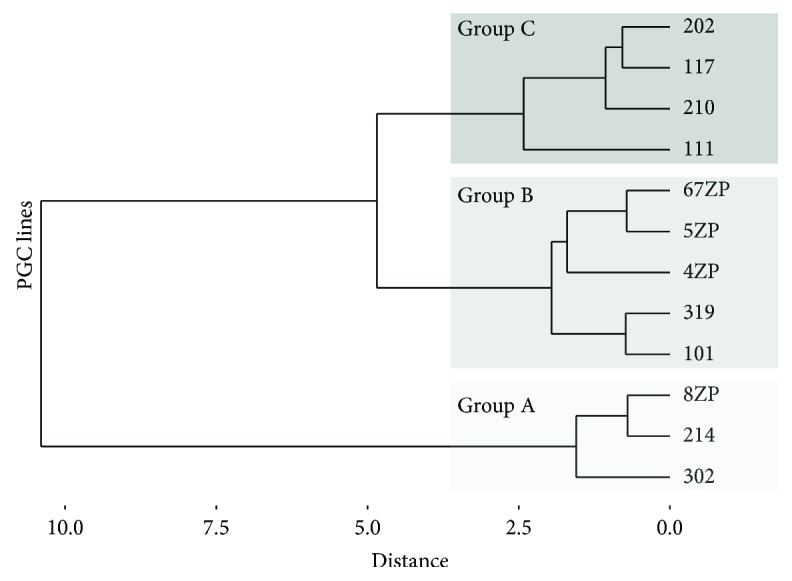
Agglomerative hierarchical clustering. Agglomerative hierarchical clustering was used to examine the similarities/dissimilarities between the PGC lines. Expression of CVH, cDAZL, cPOUV, cNANOG, and gga-miR-302a and the proliferation rate of the lines were analysed, and three groups were identified (group A, B, and C) on the dendrogram.

As a next step, agglomerative hierarchical clustering was used to examine the similarities/dissimilarities between the PGC lines. Expression of *CVH* (Figure 4(a)), *cDAZL* (Figure 4(b)), *cPOUV* (Figure 4(c)), *cNANOG* (Figure 4(d)), and gga-miR-302a (Figure 4(e)) and the doubling time (Figure 4(f)) of the lines were analysed. Three groups were identified (groups A, B, and C) on the dendrogram (Figure 5).

**Figure 6:**
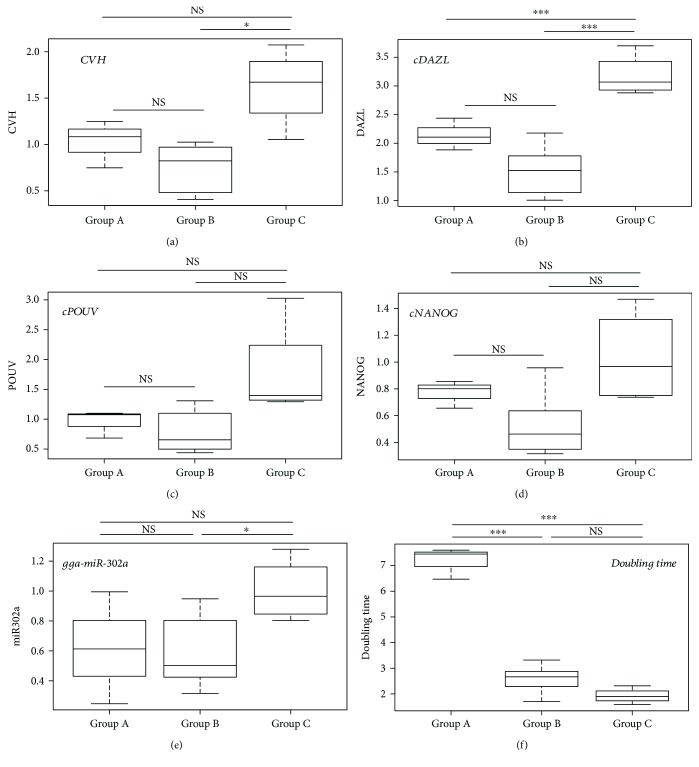
Stem cell- and germ cell-specific marker expression and the doubling time of PGCs. Expression of the germ and stem cell-specific markers in the three groups was identified by clustering analysis. The box plots represent descriptive statistical parameters (minimum, maximum, median, and first and third quartiles) which were calculated from the expression datasets for each marker. Differences among the groups identified by the hierarchical clustering were tested. Welch's *t*-tests were performed between the groups in case of each variable. Expression of the germ cell-specific markers CVH and cDAZL is shown in (a) and (b), and expression of the stem cell-specific markers cPOUV, cNANOG, and gga-miR302a is shown in (c), (d) and (e), respectively. In (f), the proliferation rate is indicated, measured as doubling time of the PGC lines. Evaluation of the *p* values: ^∗∗∗^ *p* < 0.001 and ^∗^ *p* < 0.05. NS: nonsignificant.

Differences among the groups were tested. Welch's *t*-tests were performed in the case of each variable. Expression of the germ cell-specific markers *CVH* and *cDAZL* are shown in Figures 6(a) and 6(b). Expression of the stem cell-specific markers *cPOUV*, *cNANOG*, and gga-miR302a are shown in Figures 6(c)–6(e), respectively. In Figure 6(f), the proliferation rate is indicated, measured as doubling time of the PGC lines (evaluation of the *p* values: ^∗∗∗^ *p* < 0.001, ^∗∗^ *p* < 0.01, ^∗^ *p* < 0.05, and .*p* < 0.1. NS: nonsignificant).

**Figure 7:**
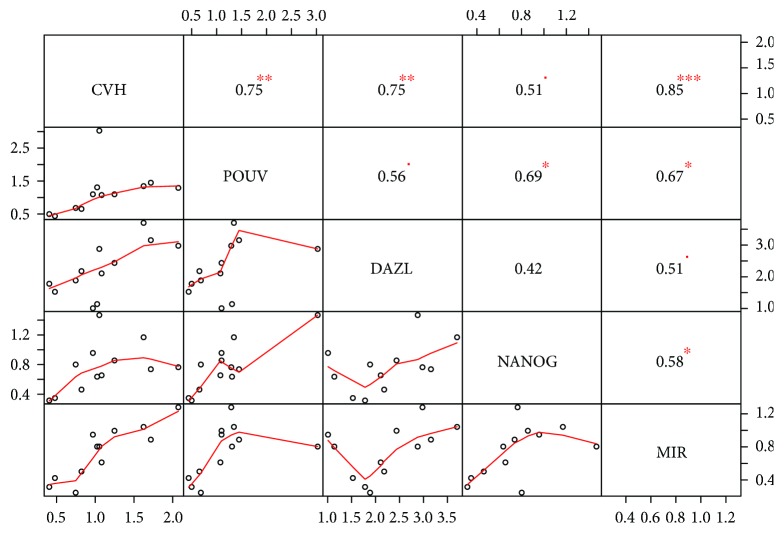
Correlation between the expression level of stem and germ cell-specific markers in the established PGC lines. Correlation coefficient (1 to −1, size of the numbers is proportional with correlation itself) and the significance of the relationship (indicated with red stars, ^∗∗∗^ *p* < 0.001, ^∗∗^ *p* < 0.01, ^∗^ *p* < 0.05, and ^▪^ *p* < 0.1) were calculated for each pair of the markers. Bivariate scatter plots are also shown with a fitted line on every marker combination.

When we checked the correlation between the stem and germ cell-specific markers, we found a high positive correlation for each marker to *CVH.* We found a high correlation ratio between the miR-302a and CVH expression as well (Figure 7).

### 3.4. Complex Microarray-Based miRNA Expression Profile Analysis in Chicken PGC Lines

**Figure 8:**
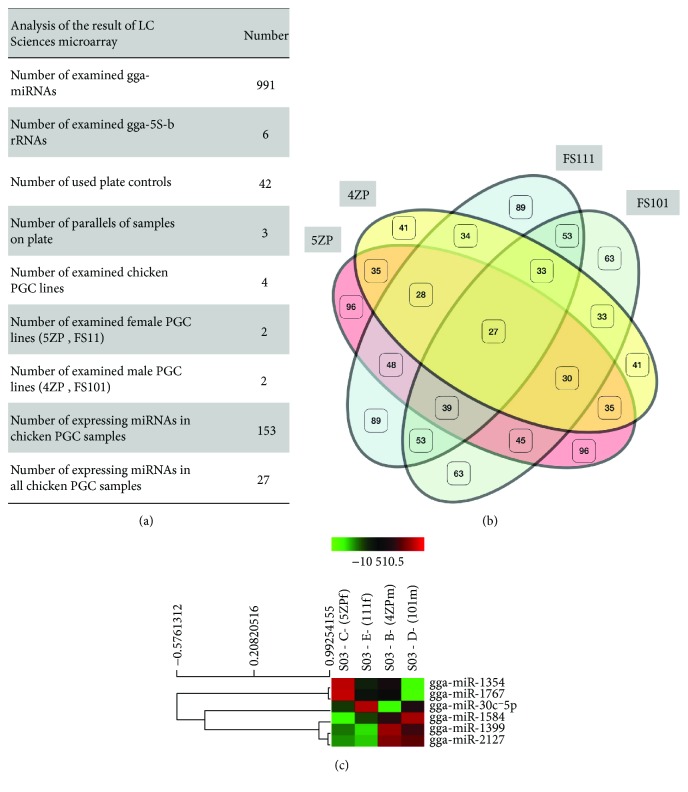
An overview of the results of LC chicken miRNA microarray analysis. (a) The microarray analysis was performed on 4 PGC lines (2 female and 2 male) (Supplementary Tables 2–5). On the microarray, 991 chicken-specific miRNAs, 42 plate controls, and 6 gga-5Sb rRNA probes were placed (Supplementary Table 6). From the 991 miRNAs, 27 miRNAs were expressed in all samples and altogether 153 miRNAs were expressed in PGC lines. (b) The Venn diagram introduces the similarities and differences between the cell lines. (c) Paired *t*-test analysis was conducted between the male (4ZP, FS101) and female (5ZP, FS111) PGC lines by LC Sciences Company. Paired *t*-test was conducted between the male and female PGC samples at the 0.05 significance level, to analyse the differences in the expression level of all expressing miRNAs in the samples. The heat map represents the result of the analysis. Only 6 differentially expressing miRNAs were found: gga-miR-1354, gga-miR-1767, gga-mir-30c-5p, gga-miR-1584, gga-miR-1599, and gga-miR-2127 (Supplementary Table 7, Supplementary Fig.  1).

As microRNAs (miRNAs) have been proved to play a key role in the maintenance of pluripotency and the cell cycle regulation of stem cells, a complex miRNA analysis (microarray-based, *μ*Paraflo® Microfluidic Biochip Technology, LC Sciences, Houston, Texas, USA) was performed to determine the difference in miRNA expression profile between 2 female and 2 male PGC lines (Supplementary Tables 2–5). Expression of 991 chicken-specific miRNAs (related to pluripotency and/or differentiation) was analysed in PGC lines (Figure 8(a)).

We compared one female (5ZP) and one male (4ZP) GFP PGC line and one female (FS111) and one male (FS101) PC PGC line using 3 parallel samples at LC microarray analysis. 27 miRNAs were commonly expressed in all 4 cell lines. The Venn diagram shows that miRNAs are expressed differentially in the PGC lines (Figure 8(b)). Paired *t*-tests were conducted between the male and female PGC samples at the 0.05 significance level, to analyse the differences in the expression level of all the expressing miRNAs in the samples. The heat map (Figure 8(c)) represents the result of the analysis. Only 6 differentially expressing miRNAs were found: gga-miR-1354, gga-miR-1767, gga-mir-30c-5p, gga-miR-1584, gga-miR-1599, and gga-miR-2127 (Supplementary Table 7, Supplementary Fig.  1).

**Figure 9:**
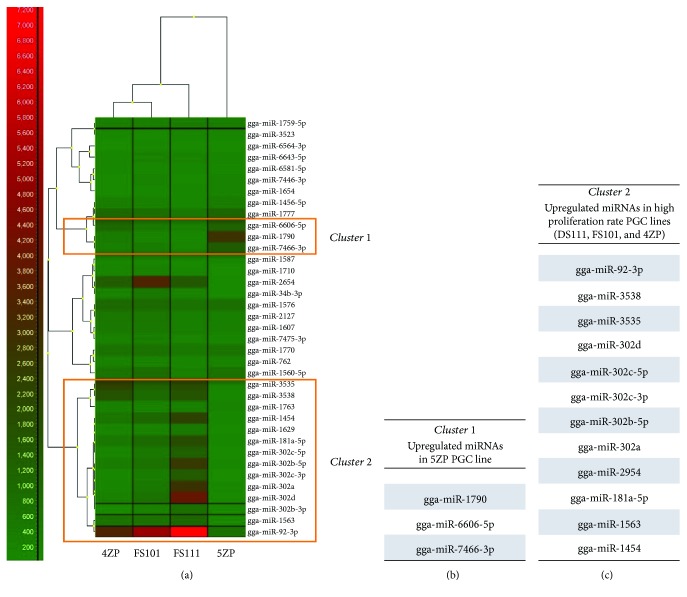
Expression pattern of miRNAs, identified in all examined samples by LC chicken miRNA microarray analysis. The microarray analysis was performed on 4 PGC lines (2 female (FS111 and 5ZP) and 2 male (FS101 and 4ZP)). (a) The expression values of miRNAs, expressing in all PGC samples, were visualized in a heat map (using GenEx software, complete linkage and Spearman correlation analysis were performed). Simultaneously, cluster analysis was performed. According to the analysis, the male cells were more related to each other. The 5ZP PGC line was the most different from the others. (b), (c) Using GenEx software, we performed a scatterplot analysis to identify the upregulated miRNAs. (b) represents the group of upregulated miRNAs in the 5ZP PGC line. These miRNAs compose cluster 1, while (c) shows the upregulated miRNAs in the highly proliferating PGC lines. These miRNAs formed cluster 2. The results of the clustering analysis highlighted a portion of miRNAs in cluster 2 which belong to the miRNA-302 cluster.

**Figure 10:**
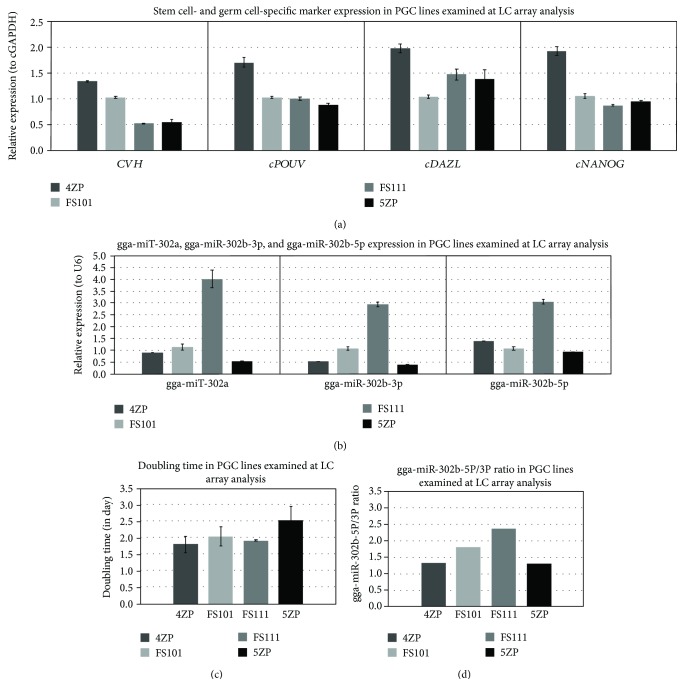
Expression pattern of stem cell- and germ cell-specific markers in RNA samples examined at LC miRNA microarray analysis. We performed qPCR analysis to check the RNA expression profile in samples sent to LC microarray analysis. We compared one female (5ZP) and one male (4ZP) GFP PGC sample, and one female (FS111) and one male (FS101) PC PGC sample using 3 parallel samples at qPCR analysis. (a), (b) Expression of CVH, cDAZL, cPOUV, and cNANOG (relative to cGAPDH as the reference gene) and gga-miR-302a, gga-miR-302b-3p, and gga-miR-302b-5p (relative to U6 as the reference gene) was analysed. Relative gene expression values were calculated relative to the FS101 sample in each case. (c) The proliferation rate of PGCs was measured on 3 different days using CCK-8 proliferation assay. The doubling time was calculated according to the measured optical densities (OD). (d) The miR-302b-5p/miR-302b-3p ratio was calculated from the average delta Ct values of samples.

Figure 9 shows the results of the heat map generated from the microarray data, reflecting miRNA expression values in the 4 PGC lines (Figure 9(a)). Using the GenEx software, we performed a scatterplot analysis to compare the expression in the male and female PGC samples and to identify the miRNAs showing maximum differential expression.  Figure 9(b) represents the group of upregulated miRNAs in the 5ZP PGC line. These miRNAs organised in one cluster (cluster 1). 5ZP showed the highest doubling time and lowest gga-miR302a, gga-302b-3p, and gga-302b-5p expression (Figure 10).  Figure 9(c) shows the upregulated miRNAs in the highly proliferating PGC lines. The results of the clustering analysis highlighted a portion of miRNAs which belongs to the gga-miRNA-302 cluster (Figure 9(c)).

**Figure 11:**
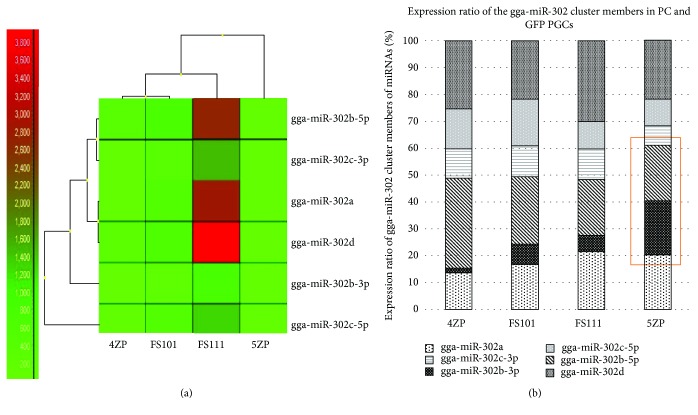
Analysis of the gga-miR-302 cluster member's expression in chicken PGCs. Microarray analysis was performed on 4 PGC lines (2 female (FS111 and 5ZP) and 2 male (FS101 and 4ZP)). (a) Expression of the members of the gga-miR-302 cluster was compared. A heat map was generated using GenEx software (complete linkage and Spearman correlation analysis were performed). (b) A high ratio of gga-miR-302b-3p expression was observed compared to gga-miR-302b-5p in the low-proliferating 5ZP PGC line. Two strands of gga-miR-302b show concordant dysregulation.

A heat map of the members of the gga-miR-302 cluster is presented in Figure 11. High expression of gga-miR-302b-5p was experienced in the highly proliferating FS111 PGC line (Figure 11(a)). High gga-miR-302b-3p expression was observed compared to gga-miR-302b-5p in the low-proliferating 5ZP PGC line. Two strands of gga-miRNA-302b show concordant dysregulation (Figure 11(b)).

We performed qPCR analysis to check the RNA expression profile in samples sent for LC microarray analysis (Figure 10). The results of the qPCR analysis and miRNA array were consistent with one another. Based on the results of the LC array analysis, the following miRNAs were selected for further detailed analysis in the future: gga-miR-302a, gga-miR-302b-5p, gga-miR-302b-3p, gga-miR-181a-5p, gga-miR-2127, and gga-miR-92-3p.

### 3.5. Transfection of Chicken PGC Lines with Anti-miR-302b-3P and Anti-miR-302b-5P Inhibitor

**Figure 12:**
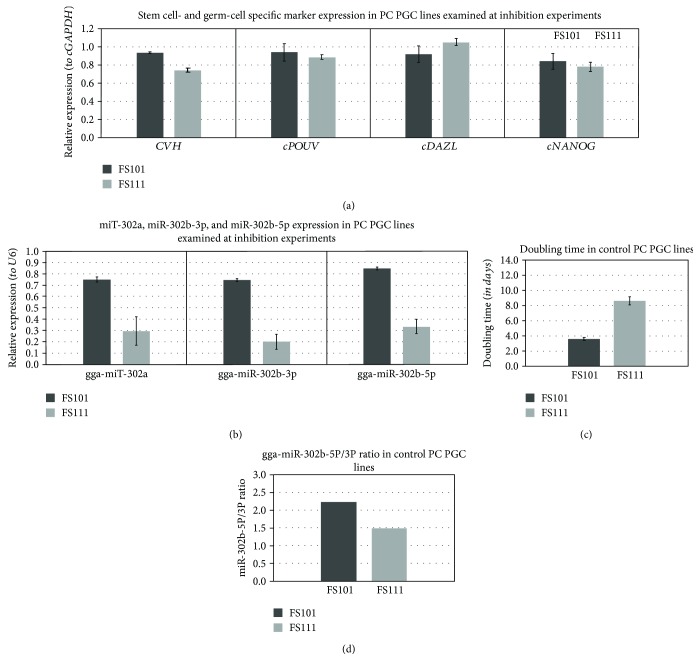
Analysis of stem cell- and germ cell-specific marker expression used at inhibition. Chicken FS101 and FS111 PC PGC lines were transfected with anti-miR-302b-5P and anti-miR-302b-3P vectors using siPORT transfection reagent. (a), (b) Expression of CVH, cDAZL, cPOUV, and cNANOG (relative to cGAPDH as the reference gene) and gga-miR-302a, gga-miR-302b-3p, and gga-miR-302b-5p (relative to U6 as the reference gene) was analysed. Relative gene expression values were calculated relative to the FS101 sample in each case. (c) The proliferation rate of the PGCs was measured on 3 different days using CCK-8 proliferation assay. The doubling time was calculated according to the measured optical densities (OD). (d) The miR-302b-5p/miR-302b-3p ratio was calculated from the average delta Ct values of samples. PC PGC line: Partridge colour Hungarian chicken embryo derived primordial germ cells.

PGCs were transfected with anti-miR-302b-5P and anti-miR-302b-3P vectors (Supplementary Table 1), at 100 nM final concentration, using a siPORT transfection agent. The proliferation rate of treated and control cells was measured using CCK-8 reagent every day at the same time, for 3 days. We analysed the proliferation rate and calculated the doubling time of control and miRNA-inhibited samples according to the measured OD values (Figure 12(c)).

PGCs were collected in lysis buffer of RNAqueous-Micro Kit 48 hours following the transfection. We performed qPCR analysis using RNA samples derived from the harvested cells. Expression of *CVH*, *cDAZL*, *cPOUV*, and *cNANOG* (Figure 12(a)) and gga-miR-302a, gga-miR-302b-3p, and gga-miR-302b-5p (Figure 12(b)) was analysed and compared with the expression ratio of gga-miR-302b-5P and gga-miR-302b-3P ratio (5P/3P) of the PGC lines (Figure 12(d)).

**Figure 13:**
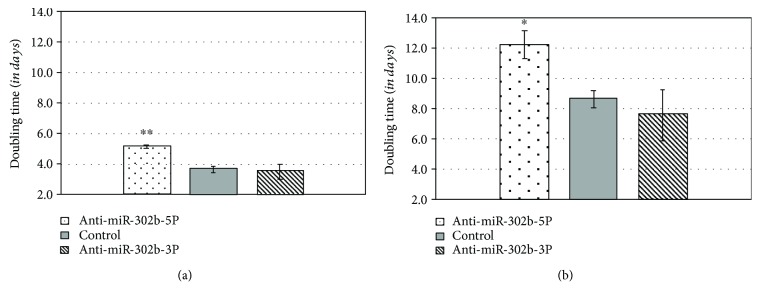
Inhibition of the gga-miR-302b-5p and gga-miR-302b-3p expression in PC PGC lines using anti-miR-302b vectors. Chicken FS101 and FS111 PC PGC lines were transfected with anti-miR-302b-5P and anti-miR-302b-3P vectors using a siPORT transfection agent. The proliferation rate of the treated and control cells was measured using CCK-8 reagent every day, for 3 days. (a) The inhibition of gga-miR-302b-5P significantly reduced the proliferation rate of the FS101 PGCs, which was revealed in a significant increase in doubling time of FS101 PC PGCs (^∗∗^ *p* < 0.001). (b) The inhibition of gga-miR-302b-5P significantly reduced the proliferation rate of the FS111 PGCs, which was revealed in a significant increase in doubling time of FS111 PC PGCs (^∗^ *p* < 0.05). PC PGCs: Partridge colour Hungarian chicken embryo-derived primordial germ cells.

We could declare that the inhibition of gga-miR-302b-5P significantly reduced the proliferation rate which appeared as an increase in the doubling time, both in the FS101 PGC line (*p* < 0.01) and in the FS111 PGC line (*p* < 0.05). The inhibition of miR-302b-3p slightly increased the proliferation rate (Figure 13).

## 4. Discussion

Results of the microarray platform revealed a large number of miRNA expressing in chicken PGCs. Some of these miRNAs were specific to individual PGC lines, and some were male- or female-specific. A paired group *t*-test analysis between the male and female samples revealed 6 differentially expressed miRNAs (*p* = 0.05). Out of 6, one of the miRNAs were identified as oncomir (gga-miR-30c-5p) and one as tumor supressor (gga-miR-2127) and one as tumor supressor (gga-miR-2127) [24]. The other miRNAs, gga-miR-1767, gga-miR-1584, and gga-miR-1599, are novel miRNAs; they were identified in a study where chickens with viral infections were examined. These miRNAs are involved in pathways controlling immune responses to viral infections: avian influenza virus and enteritis virus [25, 26]. gga-miR-1354 is a novel miRNA and needs further characterization. As these miRNAs showed differential expression in male and female PGCs, it could be assumed that this differential expression lies with the distinct morphology and development of male and female PGCs [27]. In this study, special emphasis has been put on miRNAs involved in molecular pathways regulating the pluripotency and proliferation of the chicken PGCs. The main miRNA cluster is miR-302 which is evolutionarily conserved and vertebrate-specific [15]. The main members of this cluster are miR-302a, miR-302b-3p, miR-302b-5p, miR-302c-3p, miR-302c-5p, miR-302d, and miR-367. In the microarray analysis, the four analysed PGC samples highly expressed the members of the gga-miR-302 cluster. The expression level was consistent with the published data of the miR-302 cluster being stem cell-specific miRNA [28]. However, the expression levels of miR-302b-5p and miR-302b-3p were different in the examined PGCs lines. The highly proliferating PGC lines had high expression of miR-302b-5p compared to miR-302b-3p and vice versa for the low-proliferating PGC lines (Figures 10(d) and 12(d)).

The two strands of the miRNA from the same precursor sequence were concordantly dysregulated. A study conducted by Mitra and colleagues [16] identified a concordantly dysregulated set of miRNA pairs in cancer cell lines. These pairs were either coregulated up or down or were expressive in opposite directions. The two strands had different miRNA targets, thereby regulating different mRNAs. Some of these pairs could be regulating targets involved in the same molecular pathway [28].

The members of the miR-302 cluster are involved in controlling the PGC renewal and proliferation via a mirage of transcription factors involved in important signalling pathways such as TGF*β* and MAPK signalling. The main function of the miR-302 family is to overcome the restriction point in the cell cycle, that is, G1 to S phase transition. They do this by regulating the cyclins, cyclin-dependent kinases, and other phosphatases involved in phosphorylation of the retinoblastoma protein (RB) [12].

**Figure 14:**
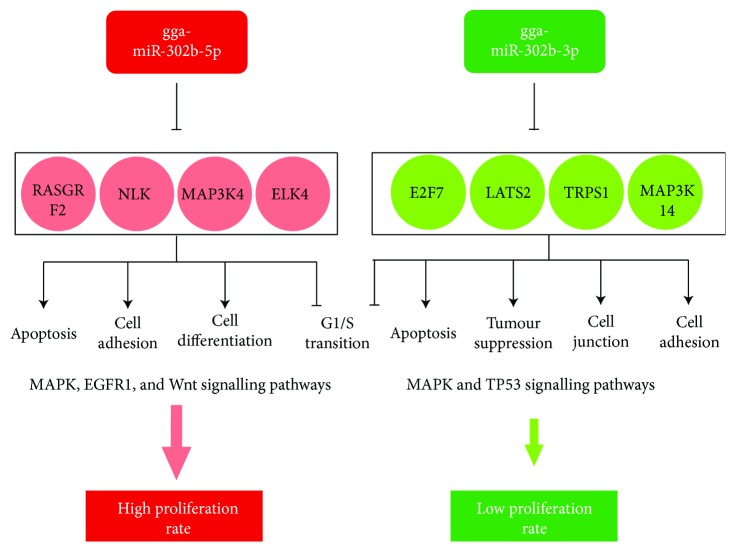
Most important targets of gga-miR-302b-5p and gga-miR-302-3p miRNAs. The main molecular targets for the studied miRNAs have been presented. The molecular pathways and targets for gga-miR-302b-5p are on the left side and for gga-miR-302b-3p on the right side. MAKP signalling is responsible for maintaining pluripotency and proliferation in mammals. It can be activated by a series of intrinsic and extrinsic stimulatory signals. In chickens, gga-miR-302b-5p controls the proliferation rate via MAPK signalling. In the case of low-proliferating PGC cell lines, gga-miR-302b-3p expression was high. It can be assumed that the high gga-miR-302b-3p expression somehow causes downregulation of pathways promoting proliferation, thereby causing cell cycle arrest at the G1 stage. gga-miR-302b-5p was found to be highly expressed in high-proliferating PGC lines. Hence, it can be hypothesized that probably the high expression of gga-miR-302b-5p is contributed in controlling throughout its molecular targets in the MAPK signalling pathway. miR-302b-5p probably by inhibiting the MAPK pathway components can cause high proliferation rate in PGC lines, and miR-302a is responsible for the fast transition from the G1 to S phase in the cell cycle [15].

Using the miRDB miRNA prediction database and the miRbase (Version 21), the main miRNA targets for both strands of the miR-302b miRNA was identified in chicken. The main molecular targets for gga-miR-302b-3p were *MAP3K14*, *E2F7*, *LATS2*, *TRPS1*, *CDK6*, *RAPGEF2*, and *TGFβR2*. For gga-miR-302b-5p, *RASGRF2*, *MAP3K4*, *NLK*, and *ELK4* are the best target genes (Figure 14). Most of them are proteins involved in the MAPK (mitogen-activated protein kinase) signalling pathway in chickens [29]. Using the KEGG database, the main molecular pathway for these targets was identified. The MAPK signalling pathway in chickens is involved in controlling the rate of cell proliferation and differentiation [30]. gga-miR-302b-5p was found to be highly expressed in high-proliferating PGC lines. Hence, it can be hypothesized that high expression of gga-miR-302b-5p is contributed in controlling the MAPK signalling pathway. miR-302b-5p, probably by inhibiting the MAPK pathway components, can cause a high proliferation rate in PGC lines, and miR-302a is responsible for the fast transition from the G1 to S phase in the cell cycle [13]. Together, taking into account the role of gga-miR-302a and gga-miR-302b-5p, both are responsible for the high cell proliferation rate but using different target molecules [12, 15]. However, the role of miR-302b-3p is in complete contrast to the role of gga-miR-302b-5p. miR-302b-3p has been cited as a tumour suppressor miRNA; that is, it suppresses proliferation via its role in the apoptosis regulation pathway. miR-302b-3p tends to suppress proliferation of human gastric cancer stem cells via the PIG3T/AKT pathway [17] (Figure 14).

Chickens and humans are physiologically and metabolically distinct. In the case of chicken's stem cells, that is, PGCs, the main molecular targets of gga-miR-302b-3p are involved in gap junction, tight junction, and adherent junction pathways [31]. It can be speculated that gga-miR-302b-3p tends to suppress cellular proliferation via contact inhibition pathways. One of the features of cellular proliferation is to lose contact with other cells for proliferation [32]; hence, in chicken probably the molecular targets of these pathways are upregulated, thereby causing low proliferation. Some of the potential targets of miR-302b-3p identified in humans (TargetScanHuman 7.1) are *MAP3K2*, *BCL6*, *CCND2*, *CCND1*, *FGF10*, *RADA2*, *SMAD2*, *PAK3*, and *TGFβR2* [13]. Their targets are also downstream targets in other molecular pathways like p53 signalling, FOXO signalling, TGF*β* signalling, and apoptosis [33].

Therefore, it can be assumed that depending upon the type of the cell line, its physiological state, and other intrinsic and extrinsic parameters, either of the strands can be activated and promote either proliferation or suppression. In slowly proliferating PGC lines, a high expression level of the gga-miR-302b-3p strand compared to the gga-miR-302b-5p strand was detected. We confirmed that the inhibition of the gga-miR-302b-5p strand significantly increases the doubling time of PGCs. We plan to perform further analysis to reveal whether the apoptosis rate is affected by the miR-302b-5p/3p ratio.

## 5. Conclusion

The LC microarray analysis revealed striking differences in the miRNA profile of male and female PGCs. There was differential miRNA expression in the PGCs based on being male- and female-specific as well as being specific to the proliferation rate. This differential expression analysis augments differences in the development, morphology, and intricate molecular pathways governing these processes. In the future, further characterization of these differential expressing miRNAs, especially gga-miR-302b-3p, gga-miR-302b-5p, gga-miR-181a-5p, and gga-miR-2127, would be done via a series of miRNA inhibition experimental analyses, with quantitative and qualitative tests to characterize the expression level of these miRNAs and their exact role, as well as mechanisms via which they influence the development rate, PGC proliferation, and pluripotency, thereby opening doors for future work like animal model system for drug or disease characterization using in vitro established PGC cultures and PGC modification for transgenic work.

## Figures and Tables

**(a):** 

PC chicken PGC line isolation	Number	%

Used embryos	74	100
Established PGC lines	21	28
Frozen PGC lines	21	28
Female cell lines	4	19

**(b):** 

GFP chicken PGC line isolation	Number	%

Used embryos	20	100
Established PGC lines	10	50
Frozen PGC lines	10	50
Female cell lines	2	20

**(a):** 

Injection of PC PGCs into recipient embryos	Number	%

Injected embryos	32	100.0
Chimera gonads	16	50

**(b):** 

Injection of GFP PGCs into recipient embryos	Number	%

Injected embryos	69	100.0
Chimera gonads	14	20

## Data Availability

All the data are provided as Supplementary Information.

## Supplementary Materials

Supplementary 1 Table 1: used primers at qPCR runs and sex PCR and inhibition.

Supplementary 2 Tables 2, 3, 4, and 5: results of LC microarray analysis.

Supplementary 3 Table 6: LC array layout.

Supplementary 4 Table 7: LC array paired *t*-test—male versus female data.

Supplementary 5 Figure 1: LC array paired *t*-test—male versus female all, heat map.
