# Supplementary figures and images for: Comparison of the MicroRNA Expression Profiles of Male and Female Avian Primordial Germ Cell Lines
Source: Stem Cells Int. 2018 Jul 10;2018:1780679. doi: 10.1155/2018/1780679 (PMC6079386; doi:10.1155/2018/1780679)

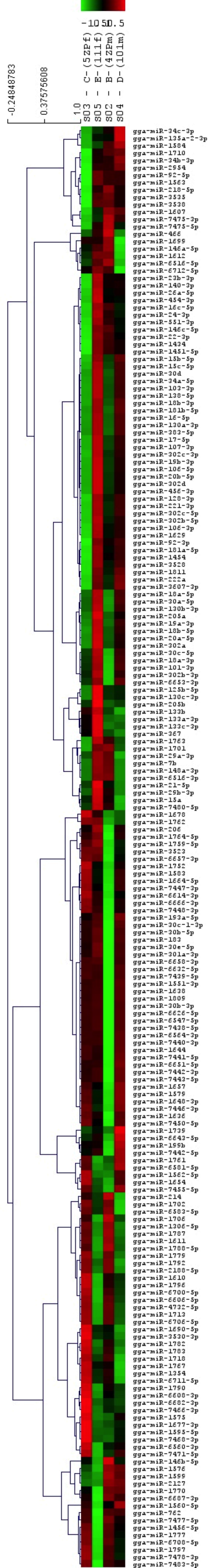

Supplement: Supplementary 5 — Figure 1: LC array paired t-test—male versus female all, heat map. [file 1780679.f5.pdf]
